# Analyses of Pharmacokinetic, Pharmacogenetic and Psychometrics Correlates of Antidepressants Use during Pregnancy and the Post-Partum Period

**DOI:** 10.1192/j.eurpsy.2022.605

**Published:** 2022-09-01

**Authors:** R. Cafaro, F. Giorgetti, L. Giacovelli, S. Vanzetto, L. Cerolini, A. Colombo, B. Dell’Osso

**Affiliations:** University of Milan, Department Of Biomedical And Clinical Sciences “luigi Sacco”, Milan, Italy

**Keywords:** psychopharmacology, Pregnancy, Perinatal psychiatry

## Abstract

**Introduction:**

About 15% of women experience a depressive episode during pregnancy, and 19% during the postpartum. Studies on safety of Antidepressants use during pregnancy have given controversial results. Obstetric-gynecological changes of pregnancy determine modifications in the pharmacokinetics of medications, through an altered metabolism of the CYP enzymes. Patient’s therapeutic response might also be influenced by polymorphisms of the genes encoding CYP enzymes.

**Objectives:**

In this perspective, we evaluated the correlation between pharmacokinetics, pharmacogenetics and psychopathological measures, analyzing SSRIs or SNRIs concentrations during the three trimesters of pregnancy, at birth and at postpartum, in order to define efficacy, tolerability and safety of Antidepressants (ADs) in the treatment of affective and anxiety disorders during pregnancy.

**Methods:**

87 patients were enrolled at the Depressive Disorders Treatment Centre (CTDD) of the Department of Psychiatry of Sacco University Hospital (Milano, Italy). Plasma concentrations of ADs were measured during first (T1), second (T2), third (T3) trimester, at birth (T4) and at postpartum (T5). Psychometric assessments were carried out. The genotype of hepatic CYP isoforms were also analysed.

**Results:**

ADs mainly metabolized by CYP2C19 (es. Sertraline) are less frequently below therapeutic range than ADs metabolized by CYP2D6. In fact, the metabolic activity of CYP2C19 is slowed down during pregnancy. The majority of ADs concentrations below therapeutic range were found in women with an accelerated metaboslism, carrier of a CYP polymorphism.

**Conclusions:**

Our results underline that the systematic use of pharmacokinetic and pharmacogenetic analyses during pregnancy could constitute a valid support in the management of therapy in the last phases of pregnancy.

**Disclosure:**

No significant relationships.

